# Student feedback experiences in a cross-border medical education curriculum

**DOI:** 10.5116/ijme.5ce1.149f

**Published:** 2019-05-24

**Authors:** Kerry Wilbur, Nawal BenSmail, Sanjida Ahkter

**Affiliations:** 1 Faculty of Pharmaceutical Sciences, the University of British Columbia, Vancouver, Canada; 2 Clinical Pharmacist, Hamad Medical Corporation, Doha, Qatar; 3 Hamad Medical Corporation, Doha, Qatar

**Keywords:** Workplace-based training, feedback, culture, pharmacy

## Abstract

**Objectives:**

To determine non-Western situated health professional student experiences and preferences for feedback in workplace-based settings.

**Methods:**

We conducted five focus groups with 27 students of Arab-origin enrolled in a Canadian-accredited cross-border pharmacy program in Qatar. Transcripts of recorded discussions were analyzed using the framework method.  Hofstede’s and Hall’s cultural dimension models were employed to understand described feedback encounters and behaviours.

**Results:**

We identified three themes associated with cultural influences on student feedback experiences, namely: 1) collectivism; 2) power distance; and 3) context.  Trainees described clinical supervisors who inadequately recognized individual performance, rejected critique, and insufficiently documented feedback onto the written in-training evaluation report. Conversely, students expected specific and timely feedback, invited criticism for learning, and desired clear written commentary.

**Conclusions:**

Feedback behaviours of clinical supervisors, but not those of trainees, were consistent with local cultural norms as described by Hofstede and Hall.  Instead, feedback expectations of pharmacy students in Qatar largely echo those of other trainees enrolled in professional curricula situated outside the Middle East. Principles for optimal feedback in clinical training largely arise from Western perspectives but are not necessarily universal. Our work demonstrates that practices, in part, may be subject to local socio-cultural influences.  This is of particular importance in the experiential training component of cross-border medical education programs adopted by overseas institutions. Our findings also further add to the growing body of literature reporting suboptimal feedback in workplace-based learning, reinforcing the need to cultivate more student-centered practices in health professional training globally.

## Introduction

Health professional training worldwide typically encompasses an experiential component whereby students acquire and consolidate knowledge and skills through participation in patient care under the supervision of clinical educators.^1^ Feedback is fundamental to enriching these experiences.  When supervisors offer information intended to improve a student’s performance compared to a standard, desirable student behaviours can be reinforced.^2^^-^^4^ Studies demonstrate feedback is most effective when it is delivered by a credible source giving specific input based on direct observation accompanied by an action plan.^5^^-^^7^

However, the principles of “good” feedback may not necessarily be universal.  What we know about feedback in clinical education is typically derived from “Western” settings.  As an example, while clinical supervisors are encouraged to offer individualized feedback in a timely fashion, there is little understanding of how these tenets might translate in different training environments.  In contexts where social harmony is emphasized, immediate corrective feedback following a task or patient encounter may be avoided given the potential loss of face.^8^ In cultures where information-sharing is not explicit, messages are communicated instead through generalizations or even non-verbal cues.^9^ Characteristics of the credible source may also vary according to prevailing norms in hierarchical structures. Study in Indonesia has illustrated how medical students and residents place the greatest value on specialist physician feedback instead of the less senior supervisors who were the most involved in their day-to-day care activities.^10^^,^^11^

Understanding feedback practices across cultural contexts are increasingly necessary. Cross-border medical education, whereby health professional programs in two countries share a curriculum, continues to grow.^12^ These partnerships typically involve institutions in Asia, Africa, or the Middle East hosting a branch or satellite campus of a “Western” university where local students can enrol and receive equivalent credentialed training.^13^ Early and ongoing experiences with these models have illustrated how curriculum developed in one context (home) may not be readily transplanted into another (host).^14^ Challenges arise when cultural relevance of delivered source content or learning and assessment behaviours of domestic students and faculty are not purposefully considered.^15^  Specific examples include how many ‘Western’ curricula offer no background information about alcohol.  Drinking is forbidden in the Muslim faith, and Arab students may have little or no exposure to alcohol consumption contributing to a practical knowledge gap in the care of patients with alcohol-related pathologies.^16^  Arab socio-cultural norms (e.g., non-confrontational dispositions) have also been attributed in part to low student participation observed in the small problem-based learning groups of Middle East programs adopting this ‘Western’ educational model.^17^  Local adaptation is now acknowledged to be requisite for successful program integration in overseas contexts, although the processes are not well described and may be at odds with measures necessary to maintain the standards of the source curriculum.

### Aim and objectives

Very little study related to the experiential training element of cross-border curricula has been undertaken.  In these direct patient health care settings, home university programs have the least influence over the quality of teaching and learning.  Host hospitals, clinics, and other practice environments are diverse and subject to their own governance and oversight infrastructures. As it pertains specifically to feedback between students and clinical supervisors, it is unclear what expectations trainees enrolled in cross-border programs carry from campus into the workplace. Similarly, to what demonstrated feedback principles, local practice educators ascribe is also poorly characterized.  The objectives of our study were to determine what are non-Western situated health professional student experiences and preferences for feedback in workplace-based training.

## Methods

### Study design

We conducted a focus group study to explore answers to our research question. An interview topic guide was developed following a comprehensive review of literature reporting other quantitative or qualitative findings and reviews about feedback in the workplace-based component of health professional curriculum (Appendix 1).

### Study setting and recruitment

Qatar is a small emirate of approximately 2 million people located in the Arabian Gulf.  With newfound affluence attributed to oil and gas revenues, Qatar has been heavily investing in its infrastructure for healthcare services and education over the past several years.  The country hosts no less than a dozen health professional training programs which either enrol students at branch campuses of their North American institutions (Weill Cornell University for medicine, University of Calgary for nursing, College of the North Atlantic for paramedicine, respiratory therapy, dental hygiene, and others) or deliver North American-accredited curricula at the national university (nutrition, pharmacy, laboratory medicine).

We recruited pharmacy students enrolled in the country’s only College of Pharmacy (CPH) at Qatar University (QU). While not affiliated with a singular, Western ‘home’ campus, the cross-border curriculum is devised to meet the standards of its Canadian-accreditation.^18^ Established in 2007, QU CPH graduates small classes of 20-30 female students annually.  Undergraduate pharmacy education is currently only offered to women at QU’s gender-segregated campus. Participants eligible for our study were students who had completed at least one experiential training course in the curriculum.  As the first workplace-based experience in this program is offered in the summer semester following the second professional year, our population included a purposive sample of third (N=24), fourth (N=25) and fifth (N=20) year students.  Potential subjects were emailed an introduction to the study and invited to participate.  We conducted five focus groups with twenty-seven interested students (6, 12, and 9, third, fourth, and fifth-year students, respectively). Ethics approval was obtained from the QU Institutional Review Board.

### Data collection

Written consent was obtained from all participants presenting to the focus group discussion, where study objectives and procedures were explained verbally and in writing. One author facilitated each audio-recoded discussion leading participants through the topic guide while a second researcher was in attendance as a field note-taker.  Participants were never addressed by name and instead assigned a participant number according to their seating assignment and to which any reported discussion quotes could be attributed.   At the end of each discussion, participants were allowed to ask additional questions or make further contributions.  Researchers also reinforced that participants respect the confidentiality of experiences and opinions expressed by members of the group. The first discussion was led by an experienced research assistant to pilot the topic guide and further model focus group facilitation to two of the authors (NB, SA), senior students in the program.  The research assistant attended the second focus group to offer additional facilitator feedback afterward.

### Data analysis

Each audio-recorded focus group discussion was professionally transcribed, and the resulting text subsequently independently verified with the original recording and finalized by one of the researchers. To further data check, these were also shared with associated focus group members for comment or possible correction. Researchers used Hall and Hofstede cultural dimension frameworks as sensitizing topics to characterize how feedback may be exchanged in this geographic setting and therefore, assume the ontological and epistemological stance of multiple socially constructed realities.  Edward Hall has delineated cultures according to the use of context and information to create meaning.^19^ Meanwhile, Geert Hofstede’s work outlines a country’s predominant cultural predisposition according to six main domains (power distance, collectivism, uncertainty avoidance, long-term orientation, indulgence, and masculinity).^20^ Relative distinctions between populations in North America and Middle East regions exist among these cultural dimensions and have in part accounted for differences found in health-related research about patient behaviours, provider care, and medical education.^21^^-^^23^ Through a framework method, two researchers deductively coded the first two focus groups separately, and then all authors convened to discuss and arrive at an initial codebook.^24^^,^^25^  The third focus group was similarly independently, and double-coded followed by group discussion and further modifications made to the codebook. The remaining two transcripts were each coded by one researcher. Coded data was charted into a matrix for purposes of summary and interpretation. Researchers examined the matrix to discuss relationships and extract themes according to our sensitizing framework and any others identified. Although we collected data until our eligible (N=69) and consenting (27, 39%) sample population was exhausted, a saturation of ideas and concepts may have been considered reached following analysis of the fourth focus group discussion as no novel codes were subsequently identified.

## Results

According to perspectives shared by participants, Hofstede and Hall's frameworks adequately characterized core themes associated with cultural influences on student feedback experiences, namely: 1) collectivism; 2) power distance; and 3) context. Expressed student preferences for feedback are included as a fourth category. Representative citations are provided for each and attributed to the specific focus group and participant.

### Cultural Influences on Student Feedback Experiences

#### Collectivism

Collectivism in Hofstede’s framework represents the extent to which individuals in a society integrate into groups.^20^ Middle Eastern populations often form tight-knit cohesive units of reciprocal care and loyalty.  Maintenance of harmony is paramount, in contrast with individualistic perspectives of many Western societies, whereby personal (and potentially conflicting) opinion is expected, and tasks prevail over relationships.

Respondents rejected individualism in their reported behaviours as they carefully considered the legacy of their feedback to supervisors, but this disposition manifested in diametric ways.  Some students were committed to offering constructive clinical site and educator feedback as they felt a responsibility to those reaching the same learning environment after them.  Conversely, a number of respondents were concerned that such candour could adversely affect the next student and censored themselves accordingly.

“I think if we give [supervisors] bad feedback they would think we are leaving a bad impression and that may be affecting other students who come later – so I’m thinking about others.” (4th year student participant 1)

When it came to input on their own performance however, students felt disadvantaged by the collectivist outlook.  Participants could not always distinguish their own evaluation from that of their classmates, making it difficult for them “to improve” and were disappointed when specific feedback of their performance was lacking. Some students identified occasions of “carry over” attitudes from a preceptor’s prior negative supervisory experience and in these instances felt cast in the same unfavourable light.  Conversely, they were demotivated when supervisors overtly minimized their abilities in contrast to others.

“One of the preceptors told me, ‘you’re not good, because the students before you were very good.’  So I started not working because, what should I do?  What should I do to impress her?” (graduate student, participant 3)

“One of the most unfair things I can ever hear – anyone can ever hear:  comparison to other students.” (4th year student, participant 4)

#### Power distance

In Hofstede’s cultural dimensions theory, power distance refers to the extent to which people in a society accept unequal distributions of power. The Middle East is a high power distance setting where those in positions of authority and seniority are respected without question, and an instructor is never contradicted or publicly criticized.^26^

Participants acknowledged how the preceptor is often viewed as the authority in local teaching and learning environments providing the backdrop for autocracy in the workplace-based assessment.  The pharmacy program in question employs an “upward feedback” process whereby a face-to-face exchange between supervisor and trainee occurs at the clerkship’s conclusion.  The student shares the written report of their learning experience with the preceptor, who in turn, reviews ratings made on the student’s in-training evaluation reports (ITERs).^27^ Although many students, typically those furthest along in the experiential training component of the curriculum, were undaunted challenging aspects of their appraisal with which they disagreed, the majority defaulted to lower positions within the perceived hierarchical structure when it came to offering feedback to their clinical supervisor.

“If she is a practising pharmacist and I am just a student, who am I to tell her that you should do this better? It’s very uncomfortable.” (3rd year student, participant 11)

“Some preceptors, they ask you, but at the same time, they expect positive feedback from you. So when you say a negative point, they take it personally.” (4th year student, participant 3)

In part, students withheld constructive feedback as they did not want to jeopardize their own evaluation. Participants were apprehensive about supervisors reading their comments and consequently also exaggerated any positive feedback.

“One of them I ranked three -, it was in the middle. So he told me, ‘Why did you rank three? We did this and that, so you still want to keep it three?’ So I felt forced to change.” (4th year student, participant 3)

“I always have to put five [“strongly agree”] so that I can get a good feedback evaluation, if I did put only four [“agree”], then I think it’ll be a big problem, so I can’t do it.” (4th year student, participant 4)

 More than one student circumvented the upward feedback process and confessed to altering their ratings on the signed report after the final evaluation and clerkship exit interview with supervisors.  The students in question conceded to the duplicity of this act but believed their particular supervisors were already disengaged in the feedback process and at the time, felt they had no other vehicle for communicating honest impressions of the training experience to the program directors.

#### Context

Context refers to the value groups place on indirect or direct communication.  For example, in Canada or the United States (low-context cultures), messages are typically explicit and in contrast to a high-context society like Qatar where communication is reliant upon non-verbal and nuanced cues.19 Information may be transmitted through an elaborate system of body language, gestures, figures or intonations of speech and employ more emotional than factual appeals.  Students in this setting sought and prioritized verbal feedback as Hall has indeed described whereby spoken agreements are preferred overwritten.  However, participants wanted sufficient detail from these conversations and the opportunity to “discuss it deeply” with their clinical supervisors.  They preferred verbal feedback as it offered the opportunity to gain elaboration on their rated performance and the opportunity to minimize misunderstandings.

“With written, you don’t see the emotions.  Like, you might take it harshly.” (4th year student, participant 12)

“Verbally, you can discuss it, you can understand why the preceptor is thinking something of you.” (5th year student, participant 13)

“I think that verbal ones like stick into your mind and give you more confidence more than written one. I feel like what they truly meant is what they say verbally.” (3rd year student, participant 3)

Most expressed an aversion to preceptor documentation of perceived negative feedback onto the written evaluation, although one participant felt that the program could learn from poorly rated student performance and make curricular improvements.  When it came to acclamatory judgments however, students were looking for detailed documentation and did not want preceptors to forget to record the specific positive points they previously shared.

“Okay, so I have good communication skills.  What does that mean?  Communication with who?  Is it with the patient? Is it on rounds?”  (4th year student, participant 7)

“When comments are written, you can look back to it and be like, yeah, that makes me feel good.” (3rd year student, participant 1)

### Student preferences for feedback

These pharmacy students outlined a number of features contributing to a positive feedback experience. Criticism was anticipated, but participants emphasized the significance of a considerate approach to advance their performance. Students, by and large, wanted to be coached.

“I really benefited when my preceptor comments on some aspects of practice I didn’t know or did wrong and they suggest what I can do to correct myself.” (4th year student, participant 6)

“When you are hearing some words like, ‘It’s okay, you are leaning.  It’s healthy to make some mistakes”, believe me, it will be effective in our avoiding these errors more than having negative feedback.” (5th year student, participant 4)

However, students were distressed when these feedback encounters were not conducted in private.  A few participants described experiences where the supervisor raised their voice in front of the care team or other students. The majority agreed they would not consider the content of feedback delivered this way, or conversely in a “joking” manner. Message credibility was also linked to the regularity of direct observation.  Participants concurred that feedback was best received from preceptors who were “with them” and “knew what they were doing”.  On the contrary, students were frustrated when principal clinical supervisors demurred judgments of un-witnessed care.  The proximity between preceptor and trainee was also conducive to the timeliness of feedback.  Participants did not want supervisors to withhold feedback and forego student opportunity to make changes before the end of the rotation.

“Always frequent feedback, and if I made a mistake now, let me know now.  Do not tell me something vague – just let me know so I will not repeat it.” (5th year student, participant 4)

“Whenever my preceptor gave me negative feedback, he would also say what are the factors that made me do that and how I can do better next time. I like this.” (3rd year student, participant 3)

## Discussion

Our findings illustrate student feedback encounters with clinical supervisors in the workplace-based curriculum are consistent with conduct anticipated by prevailing cultural values.  Pharmacy trainees in Qatar described experiences with preceptors who inadequately recognized individual performance, rejected critique and insufficiently documented feedback onto the written ITER, which corresponds with regional positions in dimensions of collectivism, power distance, and high-context communication, respectively. These results reflect prior reports of performance appraisal conduct within businesses and other organizations in this region. Face-to-face communication is favoured, workers avoid disagreement with managers and strive to please those in positions of authority who are perceived to value personal and political relationships over accomplishments.^28^^-^^30^

Indeed, while accounts of clinical supervisor feedback behaviours may be aligned with expected societal norms, pharmacy student preferences are decidedly not.  Participants in this context outlined feedback expectations that largely echo those of trainees enrolled in health professional curricula situated in the “West”: they seek specific and “in-the-moment” feedback; invite criticism for correction and learning; and desire clarity in written commentary.^7^^,^^31^ Conversely, discomfort with these feedback practices is demonstrated by international medical graduates (IMGs) from hierarchal societies, like Qatar, entering British, North American, and Australian workplaces.^23^^,^^32^^,^^33^ IMGs note they are reluctant to question supervisors for risk of appearing unknowledgeable and perceive negative performance feedback as personal.  Such discrepancy in feedback preferences among trainees from similar cultural backgrounds could in part arise from an inadvertent transfer of Western-oriented assessment practices and values from the campus-based program and into the experiential learning environment.^34^ While aspects of “hidden curriculum” are often portrayed as messages or lessons (unintended or otherwise) adversely undermining explicit teaching, our study participants describe potential positive socialization of expectations of feedback processes.35 Medical student navigation of the content in their American cross-border curriculum (e.g., clinical ethics, end-of-life care) and the realities of local practice in Qatar has also been previously described.^36^

However, sufficient data from domestic curricula exist to refute the singular impact of cross-border medical education on student experiential training feedback preferences in this Middle East setting.  Like our pharmacy trainees, undergraduate medical students and nephrology residents from native programs in Saudi Arabia sought potentially negative feedback to guide their ongoing development but most did not receive and if so, not in a timely fashion.^37^^,^^38^  Similar lack of continuous feedback demotivated students in a Jordanian nursing program.^39^ Study of clinical training in other non-Western contexts reveal Japanese medical residents feared blame (especially in front of patients or peers) for performance missteps and hesitated to consult supervisors who may be predisposed to anger.^40^ In Brazil, medical students also described dissatisfaction with instances where negative feedback communication was not private, nor accompanied by explanation or opportunity to improve.^41^ Clearly a shared set of feedback preferences can be found among health professional students internationally, irrespective of enrolment in a cross-border curriculum.  In fact, these dispositions may cross disciplinary borders. Watling and colleagues have examined common constituents of meaningful feedback in sports, music, and (non-medical) teaching and found these trainees all seek unambiguous goal-directed feedback.^42^

Our study indicates pharmacy students in this Arab cultural context exhibit feedback preferences typically associated with “Western” principles and adds to the large, long-standing and ongoing literature demonstrating health professional trainees, irrespective of geographic setting, are dissatisfied with feedback in clinical training.  Indeed, characterization of preceptor behaviours described by our participants may be made only in part through a cultural lens as a discouraging phenomenon is evident among diverse global contexts. It seems that sentiment is ‘lost in transition’ from trainee to clinical educator.  If as students, health professionals profess their expectations for fair and useful feedback encounters, why do they not, in turn, adopt compatible behaviours in their own supervisory practices?  Is it simply that the practical demands of clinical preceptorship, notably time and system constraints, overwhelm these previously inherent preferences or has a window of opportunity necessary to reinforce predilections been missed?  A glimpse into resident-as-teacher training might be telling. There is a wealth of investment in “near-peer” teaching program development to exploit house staff relationships and structure (informal, close developmental distance, small group size), but the proportional study of medical student intern perspectives on the feedback they receive from residents appears absent.^40^^-^^42^ More purposeful evaluation of educational interventions (for residents and clinicians, alike) and what organizational factors facilitate or thwart feedback culture change in workplace-based settings is needed.^46^^,^^47^  Students in longitudinal integration clerkships report positive feedback experiences as the continuity affords relationship building with preceptors who offer continuous feedback and individualize performance expectations; however, such time-extended learning conditions are not always feasible in health professional curricula.^48^^,^^49^ Means to establish short-term or “micro-environments” of trust and how these approaches might translate to other cultural contexts are relevant arenas of research to pursue.

### Limitations

Use of cultural frameworks to understand behaviours between countries or regions as we have done in our study is not without criticism. Societies are not truly monistic, and so any given individual’s conduct will not necessarily reflect described national norms and values. Hofstede’s cultural dimension theory, in particular, was initially devised through a survey of nearly 100,000 workers within a single multinational organization (IBM) in 40 countries during two finite time periods.  However, it has been widely applied in health-related research exploring cultural influences on patient behaviours, provider care, and medical education.^50^^-^^52^ As previously acknowledged, preceptor and trainee behaviours we have attributed in part to societal beliefs are also found elsewhere in dissimilar cultural environments.  For example, reservations to offering supervisors candid feedback is not unique to high power distance settings.

Additionally, this program’s upward feedback model in place at the time likely exacerbated our participants’ unwillingness to do so. Challenges to optimizing feedback conversations are multifaceted and do not lie solely with the clinical supervisor. Our work is limited to the student perspectives obtained through focus group discussions and may not wholly encompass actual behaviour as we did not take into account preceptor perspectives or directly observe encounters in this study.

## Conclusions 

Pharmacy trainees enrolled in a cross-border curriculum described feedback encounters with clinical supervisors that were aligned with the expected cultural norms of their Arab setting.  Although perspectives of international students are not widely represented in the literature, these participants shared the same feedback expectations consistently reported by “Western” situated students.  With the continued globalization of health professional education, our findings further highlight the need for program partners to purposefully explore underlying assumptions in how local instructors and students respond to the cross-border curriculum. Ongoing work to support workplace-based feedback conversations between preceptors and students is necessary, irrespective of geographic learning context.  

### Acknowledgments

This study was funded by a Qatar University internal study grant.

### Conflict of Interest

The authors declare that they have no conflict of interest.

## Supplementary material

Supplementary file 1 Appendix 1. Focus group topic guide
