# Supplementary material for: Student feedback experiences in a cross-border medical education curriculum
Source: Int J Med Educ. 2019 May 24;10:98–105. doi: 10.5116/ijme.5ce1.149f (PMC6766385; doi:10.5116/ijme.5ce1.149f)
Supplement: Supplementary file 1 — Appendix 1. Focus group topic guide [file ijme-10-93-S1.pdf]

Appendix 1

Focus Group Topic Guide

Engagement Questions

1. How do you feel clinical supervisor feedback can benefit your professional training?
2. In your opinion, what type of feedback do you consider “fair”?

Exploration Questions

3. What do you think contributes to a positive feedback experience?
4. What do you think contributes to a negative feedback experience?
5. What are your preferences for receiving a) written and verbal feedback; b) positive and negative feedback?
6. What are your experiences giving feedback to clinical preceptors?

Exit Questions

7. Is there anything you would like to add about the feedback processes or your experiences?
